# *N*,*N*-Dimethyl-3-oxo-3-(thio­phen-2-yl)propanaminium chloride

**DOI:** 10.1107/S1600536811031199

**Published:** 2011-08-06

**Authors:** A. S. Dayananda, Jerry P. Jasinski, James A. Golen, H. S. Yathirajan, B. Narayana

**Affiliations:** a Department of Studies in Chemistry, University of Mysore, Manasagangotri, Mysore 570 006, India; b Department of Chemistry, Keene State College, 229 Main Street, Keene, NH 03435-2001, USA; c Department of Studies in Chemistry, Mangalore University, Mangalagangotri, 574 199, India

## Abstract

In the title mol­ecular salt, C_9_H_14_NOS^+^·Cl^−^, the crystal packing is stabilized by weak inter­molecular N—H⋯Cl, C—H⋯Cl and C—H⋯π inter­actions, which lead to the formation of a two-dimensional supra­molecular layer which stacks along the *b* axis.

## Related literature

For the management of major depressive disorders, see: Gupta *et al.* (2007 ▶). For the dual re-uptake inhibitor drug, duloxetine [systematic name (+)-(*S*)-*N*-methyl-3-(naphthalen-1-yl­oxy)-3-(thio­phen-2-yl)propan-1-amine], see: Waitekus & Kirkpatrick, (2004 ▶). For related structures, see: Bhadbhade *et al.* (2009 ▶); Tao *et al.* (2006 ▶, 2008 ▶).
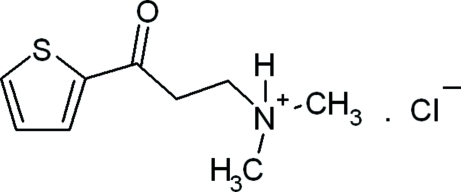


## Experimental

### 

#### Crystal data

C_9_H_14_NOS^+^·Cl^−^
*M* *_r_* = 219.72
Monoclinic, 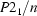

*a* = 5.8663 (3) Å
*b* = 27.0109 (9) Å
*c* = 7.1385 (4) Å
β = 110.767 (6)°
*V* = 1057.63 (9) Å^3^
*Z* = 4
Mo *K*α radiation
μ = 0.52 mm^−1^
*T* = 173 K
0.24 × 0.21 × 0.11 mm

#### Data collection

Oxford Diffraction Xcalibur Eos Gemini diffractometer
Absorption correction: multi-scan (*CrysAlis RED*; Oxford Diffraction, 2010 ▶) *T*_min_ = 0.885, *T*_max_ = 0.945
14443 measured reflections
3538 independent reflections
3290 reflections with *I* > 2σ(*I*)
*R*_int_ = 0.033

#### Refinement

*R*[*F* ^2^ > 2σ(*F* ^2^)] = 0.038
*wR*(*F* ^2^) = 0.097
*S* = 1.13
3538 reflections
124 parameters
1 restraint
H atoms treated by a mixture of independent and constrained refinement
Δρ_max_ = 0.37 e Å^−3^
Δρ_min_ = −0.28 e Å^−3^

### 

Data collection: *CrysAlis PRO* (Oxford Diffraction, 2010 ▶); cell refinement: *CrysAlis PRO*; data reduction: *CrysAlis RED* (Oxford Diffraction, 2010 ▶); program(s) used to solve structure: *SHELXS97* (Sheldrick, 2008 ▶); program(s) used to refine structure: *SHELXL97* (Sheldrick, 2008 ▶); molecular graphics: *SHELXTL* (Sheldrick, 2008 ▶); software used to prepare material for publication: *SHELXTL*.

## Supplementary Material

Crystal structure: contains datablock(s) global, I. DOI: 10.1107/S1600536811031199/tk2775sup1.cif

Structure factors: contains datablock(s) I. DOI: 10.1107/S1600536811031199/tk2775Isup2.hkl

Supplementary material file. DOI: 10.1107/S1600536811031199/tk2775Isup3.cml

Additional supplementary materials:  crystallographic information; 3D view; checkCIF report

## Figures and Tables

**Table 1:** Hydrogen-bond geometry (Å, °) *Cg*1 is the centroid of the S1/C1–C4 ring.

*D*—H⋯*A*	*D*—H	H⋯*A*	*D*⋯*A*	*D*—H⋯*A*
N1—H1*N*⋯Cl1	0.87 (1)	2.17 (1)	3.0317 (11)	171 (2)
C1—H1*A*⋯Cl1^i^	0.95	2.82	3.5641 (13)	136
C6—H6*A*⋯*Cg*1^ii^	0.99	2.97	3.8183 (13)	144

Symmetry codes: (i) 
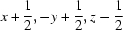
; (ii) 
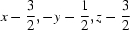
.

**Fig. 1.**
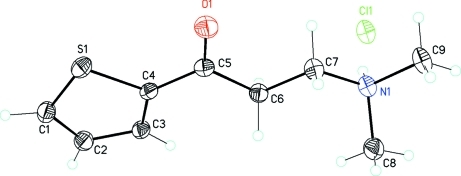
Molecular structure of the ion pair in the title compound showing the atom labeling scheme and 50% probability displacement ellipsoids.

**Fig. 2.**
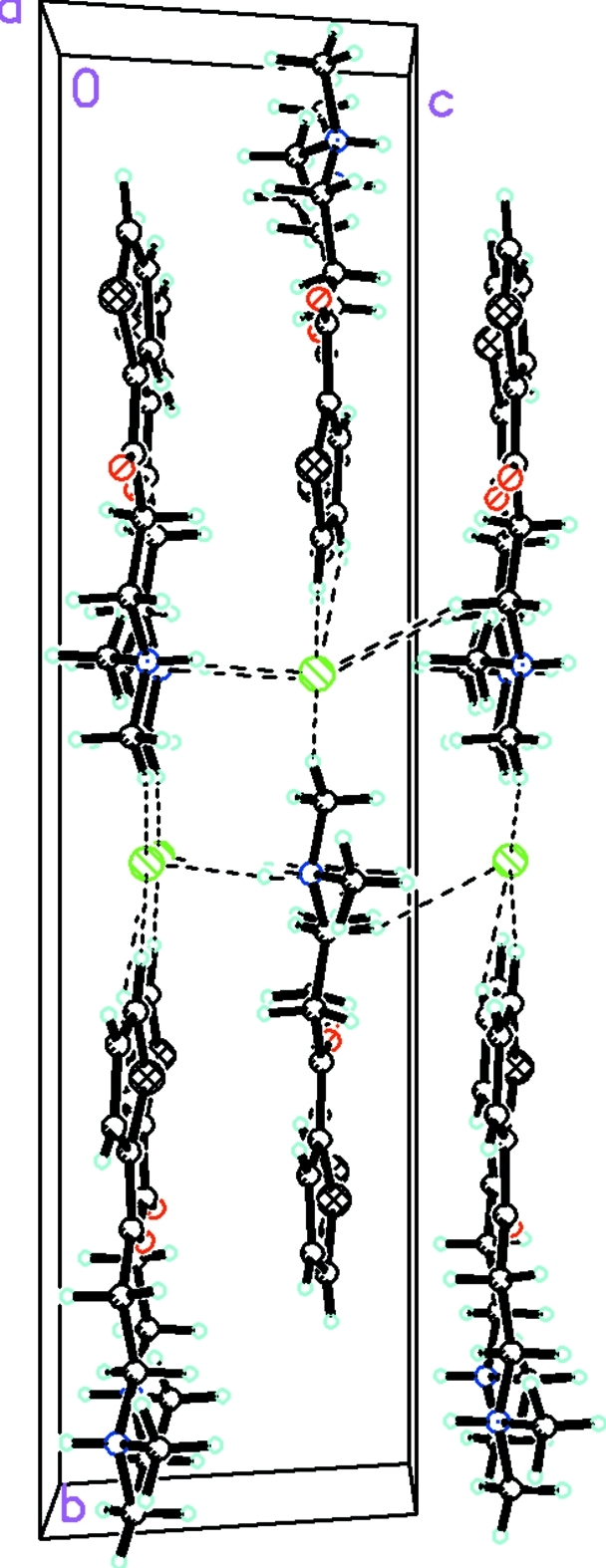
Packing diagram of the title compound viewed down the a axis. Dashed lines indicate weak N—H···Cl and C—H···Cl intermolecular interactions forming a 2-D supramolecular layer which stacks along the b axis.

## supplementary crystallographic information

### Comment

The title salt, (I), C_9_H_14_NOS^+^, Cl^-^, is an intermediate in the synthesis of duloxetine, which is a new generation drug indicated for the management of major depressive disorders as well as for neuropathic pain (Waitekus & Kirkpatrick, 2004). Duloxetine is a dual re-uptake inhibitor with actions on serotonin as well as norepinephrine (Gupta *et al.*, 2007). The crystal structures of related structures, (*R*)-3-hydroxy-*N*,*N*-dimethyl-3-(2-thienyl)-propanamine (Tao *et al.*, 2006), *N*,*N*-dimethyl-3-(1-naphthyloxy)-3-(2-thienyl)propan-1-amine (Tao *et al.*, 2008) and duloxetine hydrochloride (Bhadbhade *et al.*, 2009) have been reported. In view of the importance of duloxetine, the crystal structure of the title compound, (I), is reported.

In the molecular salt, C_9_H_14_NOS^+^, Cl^-^, one cation-anion pair makes up the asymmetric unit (Fig. 1). The crystal packing is stabilized by weak N—H···Cl, C—H···Cl and C—H..*Cg*π-ring intermolecular interactions (Table 1) forming a 2-D supramolecular layer which stacks along the *b* axis (Fig. 2).

### Experimental

The title compound was obtained as a gift sample from *R. L*. Fine chem., Bangalore. X-ray quality crystals were obtained from slow evaporation of methanol solution (*M*.pt.: 451–454 K).

### Refinement

The N—H atom was located from a difference Fourier map and refined with N—H = 0.87±0.02 Å, and with *U*_iso_(H) = 1.19*U*_eq_(N). All of the remaining H atoms were placed in their calculated positions and then refined using the riding model with C—H lengths of 0.95 Å (CH), 0.99 Å (CH_2_) or 0.98 Å (CH_3_). The isotropic displacement parameters for these atoms were set to 1.20 (CH), 1.19 (CH_2_) or 1.49–1.51 (CH_3_) times *U*_eq_ of the parent atom.

### Crystal data

**Table:**

C_9_H_14_NOS^+^·Cl^−^	*F*(000) = 464
*M**_r_* = 219.72	*D*_x_ = 1.380 Mg m^−^^3^
Monoclinic, *P*2_1_/*n*	Mo *K*α radiation, λ = 0.71073 Å
Hall symbol: -P 2yn	Cell parameters from 6745 reflections
*a* = 5.8663 (3) Å	θ = 3.1–32.2°
*b* = 27.0109 (9) Å	µ = 0.52 mm^−^^1^
*c* = 7.1385 (4) Å	*T* = 173 K
β = 110.767 (6)°	Block, colorless
*V* = 1057.63 (9) Å^3^	0.24 × 0.21 × 0.11 mm
*Z* = 4	

### Data collection

**Table:**

Oxford Diffraction Xcalibur Eos Gemini diffractometer	3538 independent reflections
Radiation source: Enhance (Mo) X-ray Source	3290 reflections with *I* > 2σ(*I*)
graphite	*R*_int_ = 0.033
Detector resolution: 16.1500 pixels mm^-1^	θ_max_ = 32.3°, θ_min_ = 3.1°
ω scans	*h* = −8→8
Absorption correction: multi-scan (*CrysAlis RED*; Oxford Diffraction, 2010)	*k* = −39→40
*T*_min_ = 0.885, *T*_max_ = 0.945	*l* = −10→10
14443 measured reflections	

### Refinement

**Table:**

Refinement on *F*^2^	Secondary atom site location: difference Fourier map
Least-squares matrix: full	Hydrogen site location: inferred from neighbouring sites
*R*[*F*^2^ > 2σ(*F*^2^)] = 0.038	H atoms treated by a mixture of independent and constrained refinement
*wR*(*F*^2^) = 0.097	*w* = 1/[σ^2^(*F*_o_^2^) + (0.0412*P*)^2^ + 0.4408*P*] where *P* = (*F*_o_^2^ + 2*F*_c_^2^)/3
*S* = 1.13	(Δ/σ)_max_ = 0.003
3538 reflections	Δρ_max_ = 0.37 e Å^−^^3^
124 parameters	Δρ_min_ = −0.28 e Å^−^^3^
1 restraint	Extinction correction: *SHELXL97* (Sheldrick, 2008), Fc^*^=kFc[1+0.001xFc^2^λ^3^/sin(2θ)]^-1/4^
Primary atom site location: structure-invariant direct methods	Extinction coefficient: 0.0190 (18)

### Special details

**Table:**

Geometry. All e.s.d.'s (except the e.s.d. in the dihedral angle between two l.s. planes) are estimated using the full covariance matrix. The cell e.s.d.'s are taken into account individually in the estimation of e.s.d.'s in distances, angles and torsion angles; correlations between e.s.d.'s in cell parameters are only used when they are defined by crystal symmetry. An approximate (isotropic) treatment of cell e.s.d.'s is used for estimating e.s.d.'s involving l.s. planes.
Refinement. Refinement of *F*^2^ against ALL reflections. The weighted *R*-factor *wR* and goodness of fit *S* are based on *F*^2^, conventional *R*-factors *R* are based on *F*, with *F* set to zero for negative *F*^2^. The threshold expression of *F*^2^ > σ(*F*^2^) is used only for calculating *R*-factors(gt) *etc*. and is not relevant to the choice of reflections for refinement. *R*-factors based on *F*^2^ are statistically about twice as large as those based on *F*, and *R*- factors based on ALL data will be even larger.

### Fractional atomic coordinates and isotropic or equivalent isotropic displacement parameters (Å^2^)

**Table:**

	*x*	*y*	*z*	*U*_iso_*/*U*_eq_	
S1	0.52202 (6)	0.201976 (12)	0.22248 (5)	0.02281 (9)	
Cl1	0.25260 (7)	0.437297 (12)	0.73047 (5)	0.02698 (10)	
O1	0.59639 (18)	0.31119 (4)	0.23658 (16)	0.0269 (2)	
N1	0.1628 (2)	0.42989 (4)	0.28618 (16)	0.0202 (2)	
H1N	0.185 (3)	0.4283 (6)	0.413 (2)	0.024*	
C1	0.3315 (2)	0.15653 (5)	0.24400 (19)	0.0232 (2)	
H1A	0.3584	0.1222	0.2307	0.028*	
C2	0.1360 (2)	0.17494 (5)	0.2825 (2)	0.0232 (2)	
H2A	0.0125	0.1549	0.3008	0.028*	
C3	0.1380 (2)	0.22742 (4)	0.29226 (19)	0.0200 (2)	
H3A	0.0157	0.2465	0.3172	0.024*	
C4	0.3377 (2)	0.24739 (4)	0.26137 (17)	0.0176 (2)	
C5	0.4056 (2)	0.29935 (4)	0.25738 (17)	0.0183 (2)	
C6	0.2273 (2)	0.33779 (4)	0.27755 (18)	0.0193 (2)	
H6A	0.0631	0.3312	0.1780	0.023*	
H6B	0.2174	0.3358	0.4129	0.023*	
C7	0.3098 (2)	0.38907 (4)	0.24408 (19)	0.0209 (2)	
H7A	0.4826	0.3934	0.3311	0.025*	
H7B	0.3006	0.3918	0.1033	0.025*	
C8	−0.1028 (3)	0.42606 (5)	0.1722 (2)	0.0301 (3)	
H8A	−0.1859	0.4550	0.2009	0.045*	
H8B	−0.1665	0.3959	0.2118	0.045*	
H8C	−0.1313	0.4248	0.0284	0.045*	
C9	0.2585 (3)	0.47836 (5)	0.2479 (2)	0.0278 (3)	
H9A	0.1668	0.5052	0.2806	0.042*	
H9B	0.2405	0.4806	0.1063	0.042*	
H9C	0.4313	0.4812	0.3315	0.042*	

### Atomic displacement parameters (Å^2^)

**Table:**

	*U*^11^	*U*^22^	*U*^33^	*U*^12^	*U*^13^	*U*^23^
S1	0.01902 (15)	0.02573 (16)	0.02610 (16)	0.00399 (11)	0.01097 (12)	−0.00015 (11)
Cl1	0.03912 (19)	0.02268 (15)	0.02328 (15)	−0.00231 (12)	0.01617 (13)	−0.00105 (11)
O1	0.0213 (4)	0.0274 (5)	0.0360 (5)	−0.0029 (4)	0.0150 (4)	−0.0012 (4)
N1	0.0250 (5)	0.0182 (4)	0.0199 (5)	−0.0021 (4)	0.0109 (4)	−0.0013 (4)
C1	0.0259 (6)	0.0205 (5)	0.0226 (6)	0.0040 (4)	0.0078 (5)	0.0007 (4)
C2	0.0221 (6)	0.0213 (5)	0.0265 (6)	−0.0012 (4)	0.0090 (5)	0.0012 (5)
C3	0.0169 (5)	0.0199 (5)	0.0243 (5)	0.0010 (4)	0.0086 (4)	0.0001 (4)
C4	0.0149 (5)	0.0197 (5)	0.0174 (5)	0.0016 (4)	0.0050 (4)	0.0003 (4)
C5	0.0173 (5)	0.0221 (5)	0.0156 (5)	−0.0002 (4)	0.0060 (4)	−0.0002 (4)
C6	0.0187 (5)	0.0196 (5)	0.0209 (5)	−0.0008 (4)	0.0086 (4)	0.0006 (4)
C7	0.0228 (5)	0.0200 (5)	0.0234 (5)	−0.0008 (4)	0.0126 (5)	−0.0007 (4)
C8	0.0240 (6)	0.0232 (6)	0.0421 (8)	0.0012 (5)	0.0106 (6)	−0.0032 (5)
C9	0.0377 (7)	0.0177 (5)	0.0315 (7)	−0.0056 (5)	0.0167 (6)	−0.0020 (5)

### Geometric parameters (Å, °)

**Table:**

S1—C1	1.7032 (14)	C4—C5	1.4620 (16)
S1—C4	1.7216 (12)	C5—C6	1.5165 (16)
O1—C5	1.2224 (15)	C6—C7	1.5139 (16)
N1—C8	1.4834 (18)	C6—H6A	0.9900
N1—C9	1.4876 (16)	C6—H6B	0.9900
N1—C7	1.4947 (16)	C7—H7A	0.9900
N1—H1N	0.870 (13)	C7—H7B	0.9900
C1—C2	1.3647 (18)	C8—H8A	0.9800
C1—H1A	0.9500	C8—H8B	0.9800
C2—C3	1.4191 (17)	C8—H8C	0.9800
C2—H2A	0.9500	C9—H9A	0.9800
C3—C4	1.3769 (16)	C9—H9B	0.9800
C3—H3A	0.9500	C9—H9C	0.9800
			
C1—S1—C4	91.69 (6)	C7—C6—H6A	109.7
C8—N1—C9	110.60 (11)	C5—C6—H6A	109.7
C8—N1—C7	114.01 (10)	C7—C6—H6B	109.7
C9—N1—C7	109.27 (10)	C5—C6—H6B	109.7
C8—N1—H1N	108.0 (12)	H6A—C6—H6B	108.2
C9—N1—H1N	107.8 (11)	N1—C7—C6	113.80 (9)
C7—N1—H1N	106.8 (11)	N1—C7—H7A	108.8
C2—C1—S1	112.38 (10)	C6—C7—H7A	108.8
C2—C1—H1A	123.8	N1—C7—H7B	108.8
S1—C1—H1A	123.8	C6—C7—H7B	108.8
C1—C2—C3	112.40 (11)	H7A—C7—H7B	107.7
C1—C2—H2A	123.8	N1—C8—H8A	109.5
C3—C2—H2A	123.8	N1—C8—H8B	109.5
C4—C3—C2	112.10 (11)	H8A—C8—H8B	109.5
C4—C3—H3A	124.0	N1—C8—H8C	109.5
C2—C3—H3A	124.0	H8A—C8—H8C	109.5
C3—C4—C5	129.24 (11)	H8B—C8—H8C	109.5
C3—C4—S1	111.43 (9)	N1—C9—H9A	109.5
C5—C4—S1	119.33 (9)	N1—C9—H9B	109.5
O1—C5—C4	121.40 (11)	H9A—C9—H9B	109.5
O1—C5—C6	121.64 (11)	N1—C9—H9C	109.5
C4—C5—C6	116.96 (10)	H9A—C9—H9C	109.5
C7—C6—C5	109.97 (9)	H9B—C9—H9C	109.5
			
C4—S1—C1—C2	−0.93 (11)	S1—C4—C5—O1	−3.55 (17)
S1—C1—C2—C3	0.86 (15)	C3—C4—C5—C6	−4.03 (19)
C1—C2—C3—C4	−0.29 (16)	S1—C4—C5—C6	175.70 (8)
C2—C3—C4—C5	179.34 (12)	O1—C5—C6—C7	6.42 (16)
C2—C3—C4—S1	−0.40 (14)	C4—C5—C6—C7	−172.82 (10)
C1—S1—C4—C3	0.75 (10)	C8—N1—C7—C6	−55.48 (14)
C1—S1—C4—C5	−179.02 (10)	C9—N1—C7—C6	−179.79 (11)
C3—C4—C5—O1	176.73 (13)	C5—C6—C7—N1	−172.73 (10)

### Hydrogen-bond geometry (Å, °)

**Table:**

Cg1 is the centroid of the S1/C1–C4 ring.

**Table:**

*D*—H···*A*	*D*—H	H···*A*	*D*···*A*	*D*—H···*A*
N1—H1N···Cl1	0.87 (1)	2.17 (1)	3.0317 (11)	171.(2)
C1—H1A···Cl1^i^	0.95	2.82	3.5641 (13)	136.
C6—H6A···Cg1^ii^	0.99	2.97	3.8183 (13)	144

Symmetry codes: (i) *x*+1/2, −*y*+1/2, *z*−1/2; (ii) *x*−3/2, −*y*−1/2, *z*−3/2.
